# MetaLab: an automated pipeline for metaproteomic data analysis

**DOI:** 10.1186/s40168-017-0375-2

**Published:** 2017-12-02

**Authors:** Kai Cheng, Zhibin Ning, Xu Zhang, Leyuan Li, Bo Liao, Janice Mayne, Alain Stintzi, Daniel Figeys

**Affiliations:** 1 Department of Biochemistry, Microbiology and Immunology, Ottawa Institute of Systems Biology, Faculty of Medicine, University of Ottawa, Ottawa, Ontario Canada; 2 Molecular Architecture of Life Program, Canadian Institute for Advanced Research, Toronto, Ontario Canada

**Keywords:** Metaproteomics, Protein identification, Quantification, Taxonomy analysis

## Abstract

**Background:**

Research involving microbial ecosystems has drawn increasing attention in recent years. Studying microbe-microbe, host-microbe, and environment-microbe interactions are essential for the understanding of microbial ecosystems. Currently, metaproteomics provide qualitative and quantitative information of proteins, providing insights into the functional changes of microbial communities. However, computational analysis of large-scale data generated in metaproteomic studies remains a challenge. Conventional proteomic software have difficulties dealing with the extreme complexity and species diversity present in microbiome samples leading to lower rates of peptide and protein identification. To address this issue, we previously developed the MetaPro-IQ approach for highly efficient microbial protein/peptide identification and quantification.

**Result:**

Here, we developed an integrated software platform, named MetaLab, providing a complete and automated, user-friendly pipeline for fast microbial protein identification, quantification, as well as taxonomic profiling, directly from mass spectrometry raw data. Spectral clustering adopted in the pre-processing step dramatically improved the speed of peptide identification from database searches. Quantitative information of identified peptides was used for estimating the relative abundance of taxa at all phylogenetic ranks. Taxonomy result files exported by MetaLab are fully compatible with widely used metagenomics tools. Herein, the potential of MetaLab is evaluated by reanalyzing a metaproteomic dataset from mouse gut microbiome samples.

**Conclusion:**

MetaLab is a fully automatic software platform enabling an integrated data-processing pipeline for metaproteomics. The function of sample-specific database generation can be very advantageous for searching peptides against huge protein databases. It provides a seamless connection between peptide determination and taxonomic profiling; therefore, the peptide abundance is readily used for measuring the microbial variations. MetaLab is designed as a versatile, efficient, and easy-to-use tool which can greatly simplify the procedure of metaproteomic data analysis for researchers in microbiome studies.

**Electronic supplementary material:**

The online version of this article (10.1186/s40168-017-0375-2) contains supplementary material, which is available to authorized users.

## Background

Microbial communities play important roles in human health [1, 2], agriculture [3], and the environment [4]. In humans, studying the composition, function, and interaction of microorganisms in samples derived from individuals during healthy and disease states holds great promises for disease diagnosis and treatment [5]. The microbial community is a highly diverse system, which makes it a challenge for comprehensive investigations. Next-generation sequencing technology can reveal the bacteria present in a microbial community. Functional gene compositions can be determined with shotgun metagenomic sequencing, although it does not provide further information on gene expression. Alternatively, metatranscriptomics and metaproteomics can be used [6]. As proteins are predominantly responsible for biological functions, acquiring qualitative and quantitative information by metaproteomics can significantly augment our understanding of microbial communities.

The applications of metaproteomics have been limited by the lack of bioinformatics tools that can handle the extreme complexity of microbial communities. In proteomics studies, mass spectrometry (MS) data from individual species can be searched against the corresponding protein database using various search engines. By contrast, in metaproteomics prior to the use of search engines, an appropriate database needs to be selected/constructed [7]. The metaproteomics data analysis workflow can be divided into three parts: pre-processing including sample specific database construction/selection; peptide identification and quantification; and taxonomy/function interpretation [8, 9]. To make this procedure simple and practical, the development of a comprehensive software platform that can meet all the requirements from pre- to post-processing is urgently needed. Some studies have started to address this problem. MetaProteomeAnalyzer provides a graphical user interface (GUI) for the analysis and visualization of data sets [10], but issues related to the database size remain unresolved. The Galaxy bioinformatics framework enables metaproteomics data analysis [11], which provides a relatively complete workflow from database generation to downstream analysis. However, advanced computer skills are required to implement this tool. Moreover, because of the additional pre- and post-processing steps, metaproteomics data analysis can be much more time-consuming than conventional proteomics studies.

The expanded search space for metaproteomic analysis leads to low sensitivity, difficulty in false discovery rate (FDR) determination, and very long searches using conventional proteomics tools [7]. The scale of the problem can be appreciated by comparing the *Homo sapiens* protein database from UniProt (www.uniprot.org) containing 159,552 proteins (7/20/2017) to the gene catalog database of the human gut microbiome consisting of 9.8 million proteins, and this number is constantly increasing (meta.genomics.cn/meta/dataTools) [12]. To solve this problem, we recently introduced the MetaPro-IQ workflow for the analysis of microbiota samples [13] which uses a reduced database. While this strategy is beneficial for peptide identification, it remains complicated and time-consuming when processing dozens of raw files.

Quantitative techniques have been used to measure the expression level of proteins in MS-based proteomics studies for over two decades [14]. Moreover, the amount of proteins from corresponding taxonomic nodes reflects the abundance of this taxon in the community and can be used to estimate microbiota diversity [15]. The simplest form of protein abundance estimation is counting the number of identified peptides from a protein and dividing the total counts by the number of theoretically observable tryptic peptides [16]. Unipept uses such a peptide counting approach for its sunburst and treeview diagrams [17] as an estimation of the relative abundance of these nodes. Generally, various types of label-free methods rely on two different strategies, spectral counts [18] or extracted ion chromatogram (XIC) intensities [19]. Extracted ion chromatogram-based methods are considered superior to spectral counting, especially for the data generated by high-resolution mass spectrometers [12, 20]. However, to the best of our knowledge, taking advantage of XIC-based label-free quantitative information of peptides to estimate the abundance of corresponding taxa, and implementing it in a fully automatic data-processing workflow, is not available in current metaproteomics software tools.

In this work, we present an integrated software platform termed MetaLab for the comprehensive analysis of metaproteomics datasets. Briefly, MetaLab provides a workflow which includes refined database generation, peptide identification/quantification and taxonomic analysis. All the steps are processed automatically in series with user intervention limited to providing the raw files. The MetaPro-IQ approach to generate the database for protein identification is integrated in MetaLab. Moreover, MS/MS spectral clustering is integrated in the workflow which reduces the processing time. Quantitative information for both peptides and taxon are provided as part of the results. As well, a matrix-based format [21] of the quantitative result can be exported, enabling the downstream analysis by other metagenomic tools. MetaLab is designed as a “one-stop” solution providing a complete, efficient, and convenient way to overcome challenges in metaproteomics data analysis. MetaLab is free for academic users and can be downloaded at http://imetalab.ca/.

## Result

### Workflow

**Fig. 1:**
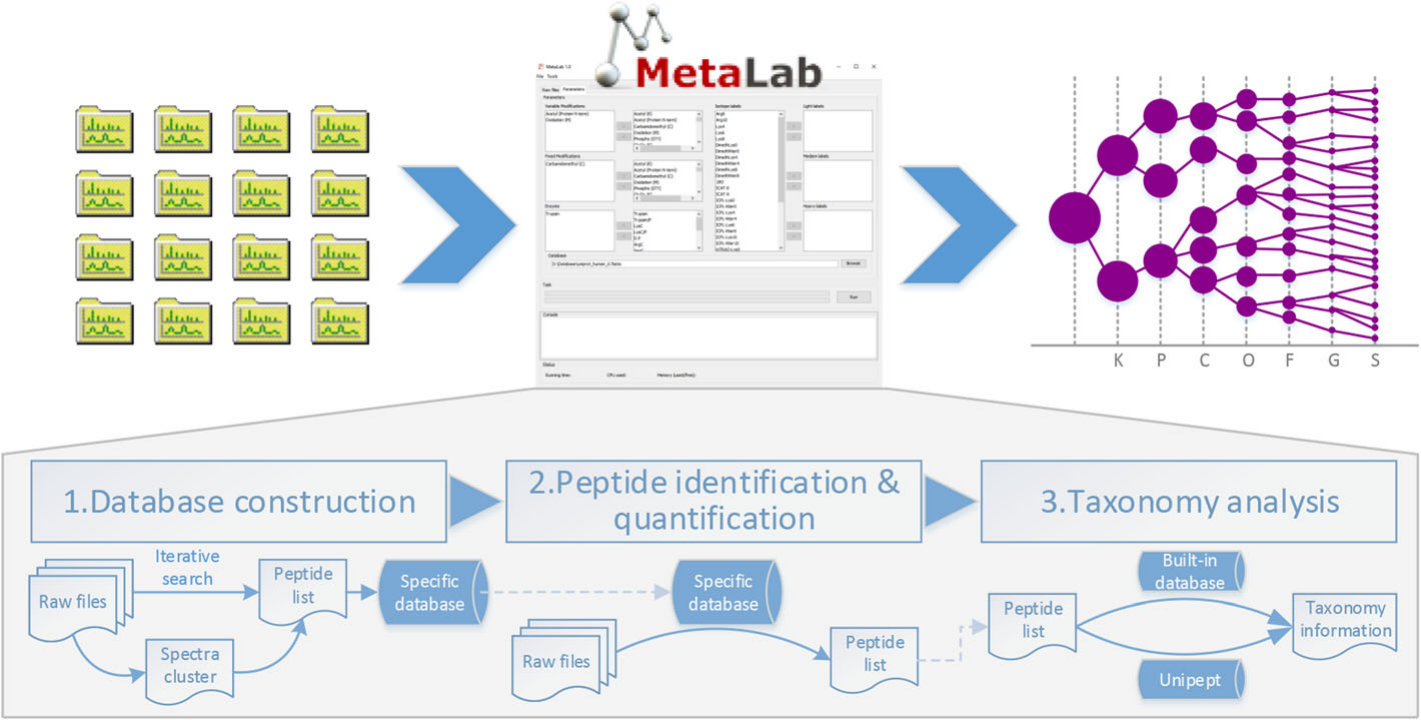
Overview of metaproteomics workflow in MetaLab. A standard pipeline consists of three parts: database construction; peptide identification; and taxonomy analysis. In database construction and taxonomy analysis steps, two alternative approaches are provided

A standard metaproteomics data analysis workflow in MetaLab contains three modules: database construction, peptide identification/quantification, and taxonomy analysis (Fig. 1). Each module and the whole workflow are performed seamlessly. Alternatively, each module can also be executed independently, enabling the users to perform customized workflows. In this section, each module in the workflow will be described in detail.

**Fig. 2:**
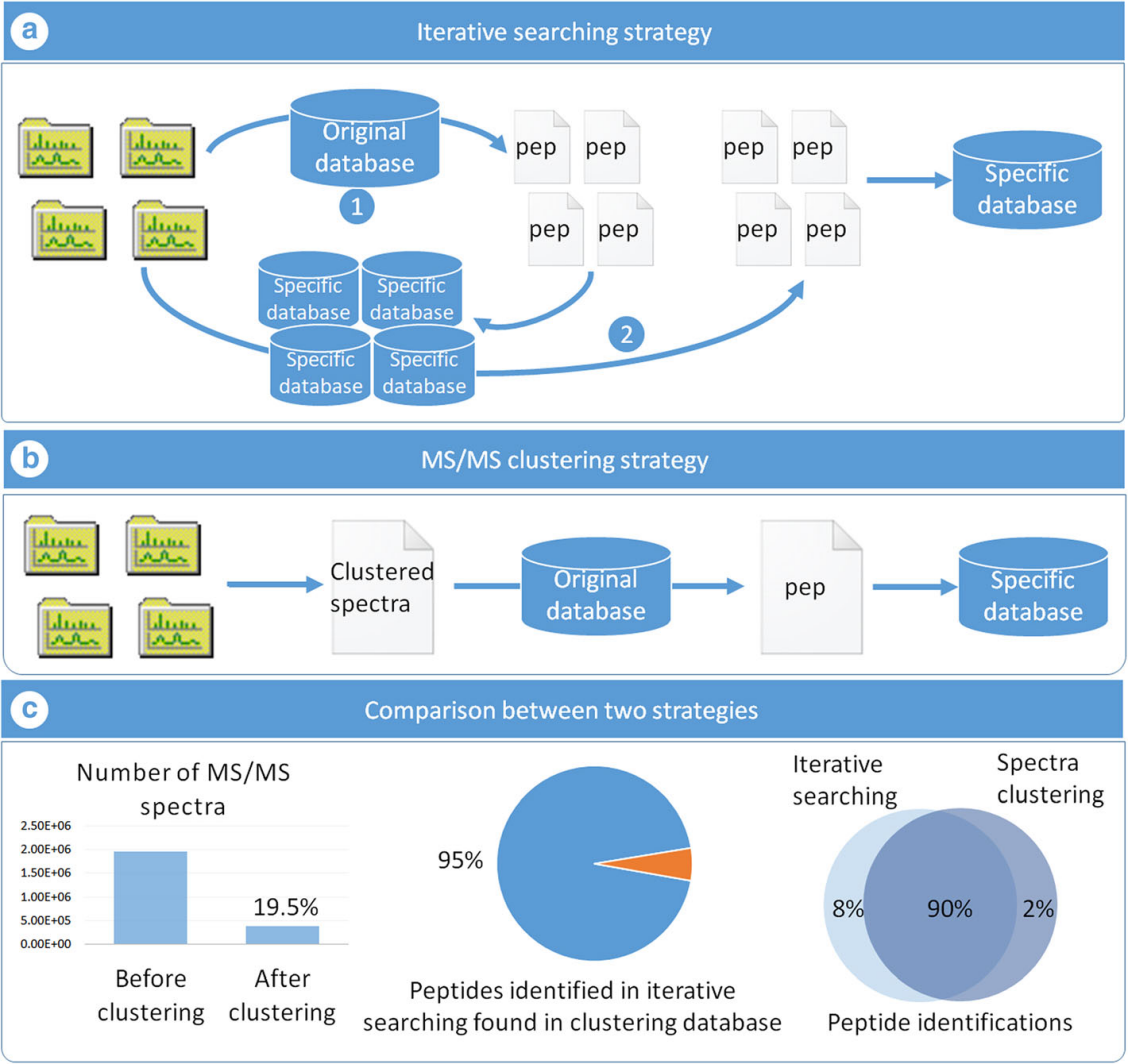
Iterative search and spectral clustering strategies used in database construction. **a** In iterative search strategy, raw files are searched against the original database separately, then for each result file the corresponding proteins are extracted to generate a specific database. All raw files are searched against the specific database once more, and the final database composed with proteins identified in the second search. **b** Firstly, a delegate spectra list is created by the clustering algorithm, then through searching these spectra against the original database, a peptide list is generated. The reduced protein database construction depends on this list. **c** Left: the total dataset contains 32 MS runs, 1,953,239 MS/MS spectra, and after clustering, only 19.5% were reserved as delegate; middle: 95% peptide identifications from iterative searching strategy were also found in database derived from clustering strategy; right: the common identifications in two strategies represent 90% of the total amount, iterative search and spectral clustering strategies only have 8 and 2% unique peptides, respectively

The function of the database construction module is to generate a reduced, sample-specific protein database from original large databases; for instance, the human gut microbial gene catalog with 9,878,647 sequences (available on http://meta.genomics.cn/) [22] or the mouse gut microbial gene catalog with 2,572,074 sequences (available on http://gigadb.org/) [23]. In this module, two approaches are available. The first approach, termed MetaPro-IQ, our previous work, implements an iterative search strategy to reduce the size of the original database (Fig. 2a) [13]. Here, MetaPro-IQ is fully automated in MetaLab. The second method incorporates spectral clustering before searching the original database to significantly reduce the processing time (Fig. 2b). The performance of this strategy is evaluated by re-analyzing the data from the MetaPro-IQ paper and details of this analysis are presented in the following section.

The second module identifies and quantifies peptides using the sample-specific database. In MetaLab, the Andromeda [24] search engine from MaxQuant [25] was adopted for the peptide characterization. Quantitative analysis can also be accomplished in this module. If the user sets isotope labels in the main frame of MetaLab, the labeling information can be written to the parameter file of MaxQuant for labeling quantification. For unlabeled samples, XIC intensity-based quantitative information are provided by a label-free quantification algorithm named maxLFQ [12] in Maxquant. By default, quantitative information is obtained by the maxLFQ algorithm.

Taxonomy analysis of the generated peptide list is performed in the last module. We create a built-in “peptide to taxonomy” database (pep2tax for short) for mapping identified peptides to the taxonomic lineages. The construction of the pep2tax database is detailed in the Methods section. Through the pep2tax database, MetaLab can match peptides to corresponding taxonomic nodes and according to the hierarchical location of the set of nodes, assign a lowest common ancestor (LCA). Abundance information of both peptides and taxa are provided according to the intensities determined in the previous module. The quantitative information of taxa at each phylogenetic rank is simply calculated as the sum intensity of all distinctive peptides of that taxon. The intensities-based quantitative information of taxa can be used to estimate the relative abundance of each taxon in one sample and to compare fold changes of the same taxon between samples.

### Evaluation of spectral clustering for the creation of specific protein databases

In MetaLab, we developed a novel strategy to increase time efficiency of database construction, by first introducing MS/MS clustering as a preprocessing procedure before the database search in metaproteomics studies. Briefly, all the MS/MS spectra are clustered according to their similarity generating a non-redundant spectra list. We adopted PRIDE Cluster ( https://github.com/PRIDE-Cluster ) into our software as it is efficient in dealing with large datasets [26]. The precursor ion mass tolerance was set as 2 Da to retain the isotopic envelope, and the fragment ion mass tolerance was set as 0.02 Da for high-resolution datasets. We also added a retention time restriction such that spectra with retention gaps exceeding 20 min will not be clustered together. The similarity threshold was set as 0.99, which represents a high similarity threshold. Following spectral clustering, the delegate spectra from all clusters are searched against the large protein database, and a peptide identification list is obtained. The sample-specific database is then constructed by extracting the protein sequence according to the peptide list and fed automatically to the next module (Fig. 2b).

Data used in the inceptive MetaPro-IQ paper was re-analyzed by the clustering strategy. This dataset contained 32 raw files, and the total number of MS/MS spectra reached 1,953,239. After clustering, only 381,834 delegate spectra remained for further analysis, representing 19.5% of the original number of MS/MS (Fig. 2c). Thus, redundant or inferior spectra, 80.5% of the total, were removed in this first step. In theory, the degree of redundancy of spectra would increase with increasing numbers of original spectra and samples. We also investigated the relationship between the original number of spectra and the factor of reduction after clustering (Additional file 1: Figure S1). For single MS runs about 20% of the spectra could be removed. However, in the PRIDE Cluster project, over 20,000,000 spectra were clustered into about 3,000,000 clusters, a nearly 85% reduction [26]. These results showed that spectral clustering could dramatically reduce the number of MS/MS spectra, especially for high-through experiments with large numbers of MS runs. Here, 1,953,239 MS/MS spectra were clustered to 381,834 in 1.5 h, which shows the great efficiency and effectiveness of this algorithm (operating environment: i7–4790 CPU @ 3.60 GHz, 16.0 GB RAM). In the original MetaPro-IQ approach, each raw file was searched twice whereas in the clustering strategy the delegate spectra were searched only once. Overall, database construction was shortened from 14 h using MetaPro-IQ to 3.7 h using the clustering strategy.

The performance of the database generated by the clustering strategy was evaluated. The size of this database (169,641 protein sequences) was slightly greater than the one created by MetaPro-IQ (157,154 protein sequences). In the MetaPro-IQ strategy, 123,927 peptide identifications were obtained, among which 95% (117,263) peptides were also found in the database generated by clustering strategy (Fig. 2c). By contrast, the database generated by the clustering strategy led to the identification of 115,832 unique peptides at a FDR below 0.01, and the commonly identified peptides accounted for 90% of the combined peptide list (Fig. 2c). We compared the peptide score distribution between these two peptide lists and overlapping curves were illustrated (Additional file 1: Figure S2). This result showed the comparable effectiveness and reliability of the two strategies for sample-specific database construction.

### MetaLab allows quantitative taxonomic analysis with metaproteomics dataset

Information on the composition and abundance of bacteria present in microbial communities is important in metaproteomics studies. Currently, Unipept is a prevalent tool that accepted a peptide list as an input to generate a table containing LCA information for matched peptides [17]. However, in their data visualizations the abundance of taxa nodes were estimated by the corresponding peptide counts. The count-based method is an improvement over purely qualitative results, but the MS signal better reflects the peptide abundance in high-resolution mass spectrometry datasets [20]. Therefore, in the taxonomic analysis step, MetaLab considers the qualitative and XIC-based quantitative information of the identified peptides to provide the taxonomic classification and abundances of bacteria in microbiota samples.

**Fig. 3:**
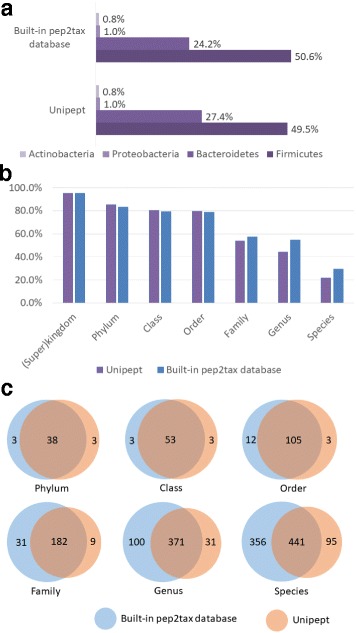
**a** The percentages of peptides assigned to four major phyla: Firmicutes, Bacteriodetes, Proteobacteria, and Actinobacteria. **b** The distribution of peptides with calculated LCAs in different phylogenetic ranks determined by built-in database and Unipept web version, respectively. **c** Overlap of taxa in each level identified by built-in database and Unipept web version

Taking the 115,832 identified unique peptides as an input, 65,407 peptides were assigned with a taxonomic lineage of LCA in MetaLab (Additional file 2: Table S1). A tree structure dataset was generated containing 2046 taxa, and for each node, the quantitative information from all samples were calculated, which can give us insight in the taxonomic diversity and comparisons between samples (Additional file 1: Figure S3). The taxonomic assignment result is in high agreement with Unipept wherein 64,717 peptides were matched (Additional file 3: Table S2), and the overlap between the two methods reached 97%. We investigated the percentages of peptides assigned to four major phyla, Firmicutes, Bacteriodetes, Proteobacteria, and Actinobacteria, and consistent results were observed (Fig. 3a). We further compared the proportion of peptides that can be uniquely assigned at each of phylogenetic rank as determined by the built-in pep2tax database and Unipept (Fig. 3b). Slightly more peptides were distinctive in phylum, order and family levels from the Unipept results. By contrast, more peptides were distinctive in the lower ranks from the built-in pep2tax result. This difference was mainly caused by the different LCA calculation strategies adopted in these two methods. In Unipept, 42% of taxonomic nodes were considered to be invalid and ignored in LCA calculations, such as species containing numbers in their names or having a parent node named *environmental samples* [27]. This cleanup aimed to remove artificial species, but a potential loss of information is also possible. Therefore in our opinion, it is appropriate to retain these types of results. The overlap of taxa identified by these two methods in each rank is shown in Fig. 3c. At the species level, 797 species were found by searching the pep2tax database, and 536 species using Unipept. Approximately 82% of the species in Unipept were also found in MetaLab. The total number of unique taxon identified in MetaLab was 48% more than that obtained in Unipept. In general, the results of taxonomic information obtained by different methods were similar. However, because of a much comprehensive usage of species for LCA calculation including those who were ignored in Unipept, more species were obtained in MetaLab.

**Fig. 4:**
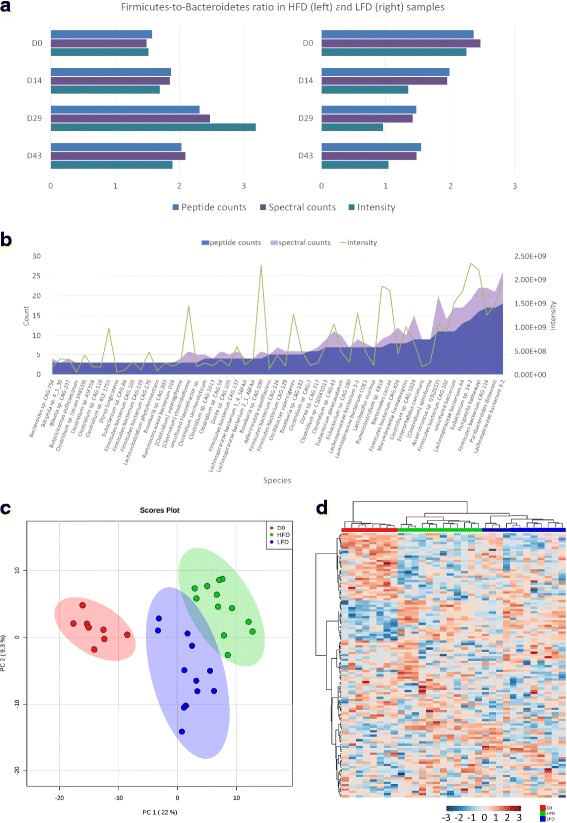
**a** Bar chart of Firmicutes-to-Bacteroidetes (F/B) ratios in HFD (left) and LFD (right) samples calculated by peptide counts, spectral counts and XIC intensity. After days of feedings, the ratios in HFD samples increased significantly. **b** Comparison of peptide counts, spectral counts and intensities of 57 species in sample HFD2_29 (nomenclature detailed in Methods). **c** PCA score plot of taxon in species level among 32 samples. Red: initial condition; blue: LFD feeding; green: HFD feeding. **d** Heat map of intensities of high confident species in 32 samples. Hierarchical raw clustering was performed by Log_10_ (intensity). Red: initial condition; blue: LFD feeding; green: HFD feeding

We also compared the quantitative results of taxa based on peptide counts, spectral counts, and XIC intensities, which were calculated by summing up the unique peptide counts, the MS2 spectra counts, and intensities, respectively. We calculated the abundance ratios between two phyla, FIRMICUTEs and Bacteroidetes, through peptide counts, spectral counts, and intensities from mouse stool samples following up to 43 days of feeding with high-fat versus low-fat diets (HFD and LFD, respectively; Fig. 4a). An increase of Firmicutes-to-Bacteroidetes (F/B) ratio was observed in HFD samples using all three methods, and the trend was stronger when ratios were determined using intensities. Abundance ratios for taxa that had numerous peptides and spectral counts were nearly identical. By contrast, count-based strategies were less sensitive for the determination of the relative abundance of species with few identification counts. For example, the abundance values of 57 species with unique peptide counts from 3 to 20 from sample HFD2_29 (nomenclature of samples detailed in Methods) were illustrated in Fig. 4b. Although it appeared that some species did not change when using peptide or spectral counts, the quantitative information derived from peptide intensities revealed significant changes. Therefore, peptide intensity-based approach provides higher sensitivity when calculating relative abundance of species, particularly when dealing with lower abundance species. Finally, we used the XIC intensity-based quantitative information for statistical analysis at the species level. In principal component analysis (PCA), three clusters corresponding to day 0 (initial condition), HFD feeding, and LFD feeding samples were obtained (Fig. 4c). We also performed cluster analysis among the 32 samples. To improve the accuracy and reduce the impact of missing values, only species with high confidence were selected. If a species had equal to, or greater than three unique peptides in one sample, the species was “valid” in this sample. Species who were valid in more than eight samples were selected for the hierarchical cluster analysis. Finally, 150 species were used and explicit group results between LFD and HFD samples were obtained (Fig. 4d). Samples from the initial condition were grouped together. After days of feeding, the samples with different diets were evidently separated into different clusters. The classification result was better than that acquired by count-based approaches (Additional file 1: Figure S4), which confirmed the value of XIC intensity-based label-free quantitative strategy in metaproteomics.

### Generic type output format for post-analysis

To further facilitate the analysis of metaproteomics dataset by biologists, a BIOM (Biological Observation Matrix, http://biom-format.org/) [21] format result file was also exported. In this format, a table was used to store the amount of biological observations from all the samples. For example, in metagenomics, the observation is the taxa identity and the corresponding value is the count of reads. In metaproteomics, the intensities of MS signal were used to replace the counts of reads representing the abundance of the specific taxa. Through this, the data generated by MetaLab can be directly subjected to downstream analysis by tools supporting this format, such as MEGAN [28], a well-known metagenomics software providing sample comparing functions. A notable merit of MEGAN is that various visualization techniques are available to show the comparison between samples. By importing the .biom format result file, various types of charts were obtained that can be beneficial during the interpretation of the results (Additional file 1: Figure S5).

## Discussion

We have introduced MetaLab, a software which aims to provide a comprehensive bioinformatic workflow for metaproteomics data analysis. MetaLab provided an intuitive GUI which greatly facilitated the procedure of data processing (Additional file 1: Figures S6–S8). A previous established workflow MetaPro-IQ which showed great performance for microbial protein identification was incorporated in MetaLab. Additionally, the new features in MetaLab made it more appropriate for the analysis of big datasets. In the MetaPro-IQ approach, all raw data are first searched against the original database and then all identified proteins are extracted to construct a refined database, which works well to reduce the size of the database, but slows the workflow. In most cases, samples share a considerable number of peptides, and peptides in high abundance generate multiple MS/MS spectra, which result in repeated peptide spectra in different or even the same MS raw files. Once a peptide is identified in one sample, the corresponding proteins will be recorded, so the identification of the same peptide repeatedly in other samples is redundant. Theoretically through selecting one delegate spectrum from similar spectra and subjecting instead only this single representative spectrum to database search, the speed of peptide identification will be improved. Here, we employed the MS/MS clustering algorithm in the database construction step, which efficiently reduced the total number of MS/MS spectra to about 20%. The peptide identification results of these two approaches were similar, but the processing time was shortened by 74% in the clustering strategy.

Investigating the quantitative profile of microbial species is an emerging field in metaproteomics [29]. In Unipept, the quantitative information is calculated based on the peptide numbers [17]. In this case, the incorporation of XIC-based abundance information for each peptide for more accurate taxonomic profiling needs further manual calculation. By contrast, both qualitative and quantitative information were integrated in the MetaLab pipeline, which allows the peptide XIC intensity information from different samples to be used to calculate the abundance of a specific taxa. In the LCA calculation, a relatively conservative strategy was adopted in MetaLab. In Unipept the species containing a number in their names were ignored in LCA calculation [27]. These species were retained in MetaLab to reduce the risk of losing useful information. In particular, in the human microbiome, there are many microbial species that have not been well characterized and therefore do not have formal names. Because of the difference in calculation methods, 48% more species were obtained using MetaLab rather than Unipept. In total 2046 taxa were quantitatively identified from 32 samples. The comparison of different quantitative strategies performed in this work confirmed the value of XIC based technique in metaproteomics. Nevertheless, investigating the quantitative composition of microbial ecosystems still remains a challenge. For example, the abundance of each taxon was measured by peptides uniquely attributed to this node. The peptides shared by other species were not considered because the quantitative contribution of the shared peptides was indistinguishable. To obtain accurate quantitative information of taxon, how to improve the precision of taxonomic assignment still needs to be resolved.

## Conclusions

Metaproteomics is a promising technology for functional analysis of microbial communities. However, interpretation of the data still remains a challenge for researchers. MetaLab allows users to get both the peptide and taxa abundance information from their raw MS data directly and automatically. Firstly, MetaLab enabled the generation of a sample-guided reduced protein database from large gene catalog databases. Identifying peptides from this reduced database can significantly improve the efficiency and reliability. Moreover, a novel strategy introducing MS/MS clustering as a preprocessing procedure before the database search was incorporated for the first time in a metaproteomics study. This strategy dramatically improved the processing speed without loss of sensitivity and accuracy. Secondly, both peptides and taxa are exported with the information of abundance giving researchers a quantitative profile of the samples. From this result, statistical analysis can be easily accomplished either at the peptide or taxon level. Finally, the result file can be exported and used for post-analysis and visualization in other widely used software tools in metagenomics such as MEGAN. Taking advantage of the workflows in MetaLab, conventional processing steps such as analyzing each data file step-by-step manually, switching on different tools, or converting data format for input/output are no longer needed for metaproteomics data analysis.

## Implementation

MetaLab is developed in Java, a platform independent language, so it can be executed on any platform with a Java Runtime Environment (JRE). MetaLab integrates several open source third-party libraries: MzJava is a versatile tool for MS data analysis and the functions concerning spectrum processing and protein digestion are used in MetaLab [30]. Spectral clustering is performed by PRIDE Cluster [26, 31], which is an open source algorithm used in PRIDE database [31]. MetaLab uses SIGAR (https://github.com/hyperic/sigar) to display the system information such as utilization of CPU and memory. Database search engines Andromeda [25] and X!Tandem [32, 33] are involved for peptide identification. Users need to download MaxQuant separately (due to license terms) and implement a simple configuration following the detailed instructions in our manual. Msconvert [34] is adopted for the conversion of MS spectra data format. MetaLab supports both GUI and command line operation modes. The parameter settings can be saved as a single parameter file which adopts JSON format for the convenience of human reading and data exchange. MetaLab can also be executed on command line through the compiled parameter file making it easily incorporated with other data-processing workflows. The online version is under development.

MetaLab is readily available after being downloaded without need for installation. It occupies 32.6 GB disk space, including a “peptide to taxonomy” database, which accounts for 32.6 GB. MetaLab could also run without the “peptide to taxonomy” database, and in this case the taxonomy analysis can be processed in Unipept API mode. Quad-Core processors with speeds of 3.0 GHz are recommended. Typically 8 GB of memory is required while the application is running.

In the main frame of MetaLab, there are two tabs, “Raw files” and “Parameters”. In “Raw files” panel users can add raw files and set the experiment names, and specify the output directory for the results (Additional file 1: Figure S6). In “Parameters” panel, parameters such as variable modifications, fixed modification, enzymes, isotope labels, and the database used for protein identification can be set (Additional file 1: Figure S7). After these settings are complete, users can start the task by a click of the “Run” button. The progress bar will show the progress rate and the text field will show the status of the processing. After the task is finished, three folders will be generated in the result directory, including “database”, “parameter”, and “result”. The constructed sample-specific database will be saved in the “database” folder. Database search engines are used in the database construction and peptide identification steps, and the corresponding parameter files are copied to the “parameter” folder for further check. The log file containing the task property and history is also saved to the “parameter” folder. There are three folders in the “result” directory: “pre_processing”, “pep_iden” and “taxonomy”. The clustered spectra list and corresponding identification results are stored in the “pre_processing” folder. The peptide identification results from the sample specific database are saved in “pep_iden” folder. In the “taxonomy” folder, information including identified peptides and taxa are stored in a XML format file, and a human readable table file is exported in xlsx format. Additionally, a biom format file is saved for the downstream analysis of this data set, which can be used by other tools such as MEGAN.

The result file exported by MetaLab contained two parts: peptide list and taxa list. In the peptide list, the following attributes were provided for each peptide: the identification information from Maxquant containing the score, PEP value and number of missed cleavages; the taxonomic lineage of LCA; the MS2 spectra counts and MS1 peak intensities in different samples. In the taxa list, all possible organisms were listed, including the identified LCAs and their ancestors, which can be used to generate a complete taxonomic tree for this dataset. The MS2 spectra counts and XIC intensities were calculated by summing up all the corresponding peptides attributed to this taxon.

The setting panel of MetaLab was used to specify customized workflows and set basic parameters (Additional file 1: Figure S8). Users can choose to partially or completely perform the three steps in the workflow: sample-specific database construction; peptide identification/quantification; taxonomy analysis. Alternative strategies for step 1 and step 3 were provided so users need to determine which method should be used. For the instrument resolution there were two options, “high-high” means both MS1 and MS2 were performed in high resolution mass analyzer (e.g., Orbitrap) and “high-low” means MS1 was taken in high resolution mode and MS2 was taken in low resolution mass analyzer (e.g., ion trap). If no change is made, MetaLab will execute using the parameters specified the previous time it was used.

### Construction of the pep2tax database for taxonomy analysis

This database was configured based on the entire UniProt protein dataset (http://www.uniprot.org/). The UniProtKB database may contain redundant protein sequence, so we utilized the non-redundant database UniParc (http://www.uniprot.org/uniparc/) for in silico digestion. The corresponding UniProtKB IDs can be found for each entry in UniParc, which were used for retrieving the information about taxonomic lineage IDs of this protein. Totally, 128,524,116 protein entries were in silico digested by trypsin with a maximum of two missed cleavage sites. Peptides with less than 6, or more than 35 amino acids, were discarded. Finally 2,071,800,323 unique peptide records were complied with the sets of taxonomic information from the corresponding protein entries to form the pep2tax database used to automatically perform taxonomic analysis in MetaLab. The size of the pep2tax database is 32.6 GB and can be downloaded separately from the core of MetaLab. MetaLab can also be run without the pep2tax database. The Unipept API has been integrated in MetaLab. The taxonomic analysis can instead be performed using Unipept API automatically. Because miss cleavages were not considered in Unipept API, less taxa will be obtained in this mode. The users can choose one or both of the two methods to perform the analysis in this module. The formats of the resulting files from both methods are uniform.

### Analyzing metaproteomics data from mouse gut microbial samples in MetaLab

The detailed information of samples and experiment design was described in our previous work [13]. The name of each sample consists of three pieces of information: diet type, mouse ID and the number of days on diet when sample was collected. For example, LFD2_D29 means the ID of the mouse is 2, fed with low-fat diet and the fecal samples were collected at day 29 on diet.

The original mouse gut microbiota protein database was downloaded from GigaDB (http://gigadb.org/dataset/view/id/100114/token/mZlMYJIF04LshpgP) [23]. The metaproteomics data was generated on a Q Exactive mass spectrometer (ThermoFisher Scientific Inc.) so the instrument resolution option “high-high” mode was selected in MetaLab. Other parameters contained: carbamidomethylation of cysteine as a fixed modification; oxidation of methionine and acetylation of protein N-terminal as variable modifications; trypsin as enzyme with maximum of two missed-cleavages. In the peptide identification step the FDR was set as 0.01.

### Statistical analysis

PCA and hierarchical clustering analysis were performed by an online tool named MetaboAnalyst 3.0 (http://www.metaboanalyst.ca/) [35]. After the submission of datasets, missing value estimation was applied and k-nearest neighbors algorithm (KNN) was used in this step. In the sample normalization step, the data were normalized by median, then transformed to logarithm format. In the cluster analysis, the distance was measured by Pearson correlation.

## Additional files

Additional file 1: Figure S1. In spectral clustering, the reduction rate of MS/MS spectra will increase with the growth of number of raw files. Figure S2 The distributions of peptide scores in iterative searching strategy and spectral clustering strategy. Figure S3 The quantitative profile of the phylogenetic tree dataset. Figure S4 Heat map of (A) peptide counts; (B) spectral counts of bacterial species in 32 samples. Figure S5 Charts illustrate the taxonomy profiles between different samples. Figures S6–S8 The GUIs of MetaLab. (DOCX 1319 kb)

Additional file 2: Table S1. List of LCAs calculated by MetaLab built-in database module. (XLSX 17593 kb)

Additional file 3: Table S2. List of LCAs calculated by Unipept. (CSV 9146 kb)

## Abbreviations

BIOM: Biological observation matrix
FDR: False discovery rate
GUI: Graphical user interface
HFD: High-fat diet
LCA: Lowest common ancestor
LFD: Low-fat diet
MS: Mass spectrometer
PCA: Principal component analysis
XIC: Extracted ion chromatogram
