# Supplementary material for: MetaLab: an automated pipeline for metaproteomic data analysis
Source: Microbiome. 2017 Dec 2;5:157. doi: 10.1186/s40168-017-0375-2 (PMC5712144; doi:10.1186/s40168-017-0375-2)
Supplement: Supplementary file 1 — In spectral clustering, the reduction rate of MS/MS spectra will increase with the growth of number of raw files. Figure S2 The distributions of peptide scores in iterative searching strategy and spectral clustering strategy. Figure S3 The quantitative profile of the phylogenetic tree dataset. Figure S4 Heat map of (A) peptide counts; (B) spectral counts of bacterial species in 32 samples. Figure S5 Charts illustrate the taxonomy profiles between different samples. Figures S6–S8 The GUIs of MetaLab. (DOCX 1319 kb) [file 40168_2017_375_MOESM1_ESM.docx]

# MetaLab: an automated pipeline for metaproteomics data analysis

Figure S1. In spectra clustering, the reduction rate of MS/MS spectra will increase with the growth of number of raw files. It can be considered that at least 20% redundant spectra will be removed after spectra clustering. At most about 85% of spectra can be deducted if vast number of spectra were processed.


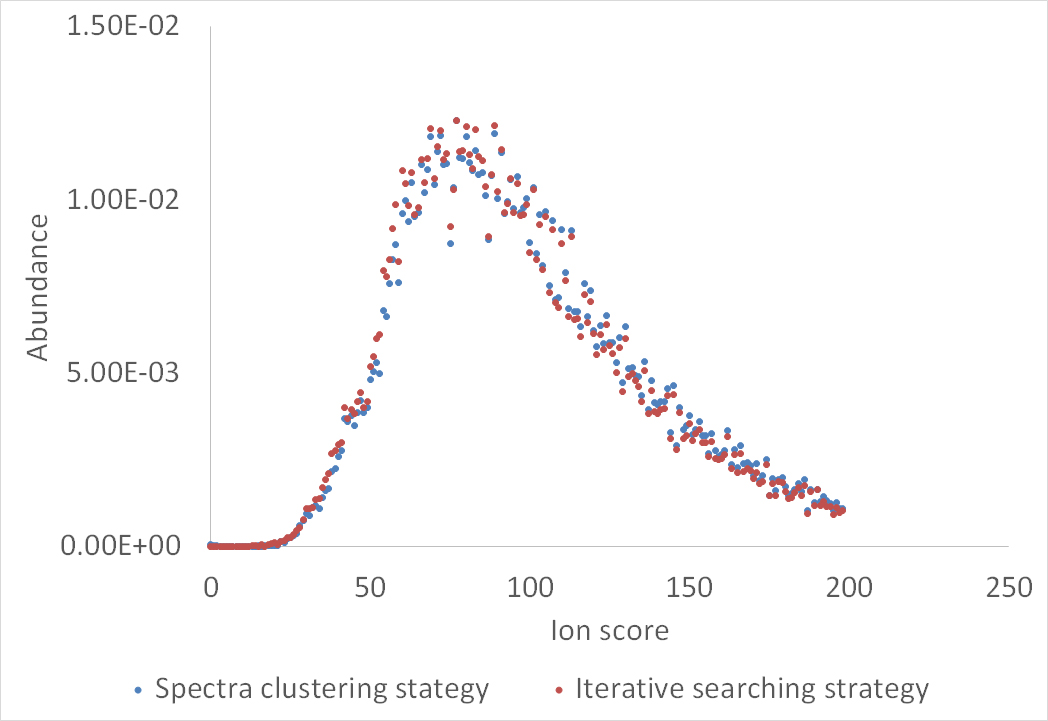


Figure S2. The score distributions of peptide scores in iterative searching strategy and spectra clustering strategy.


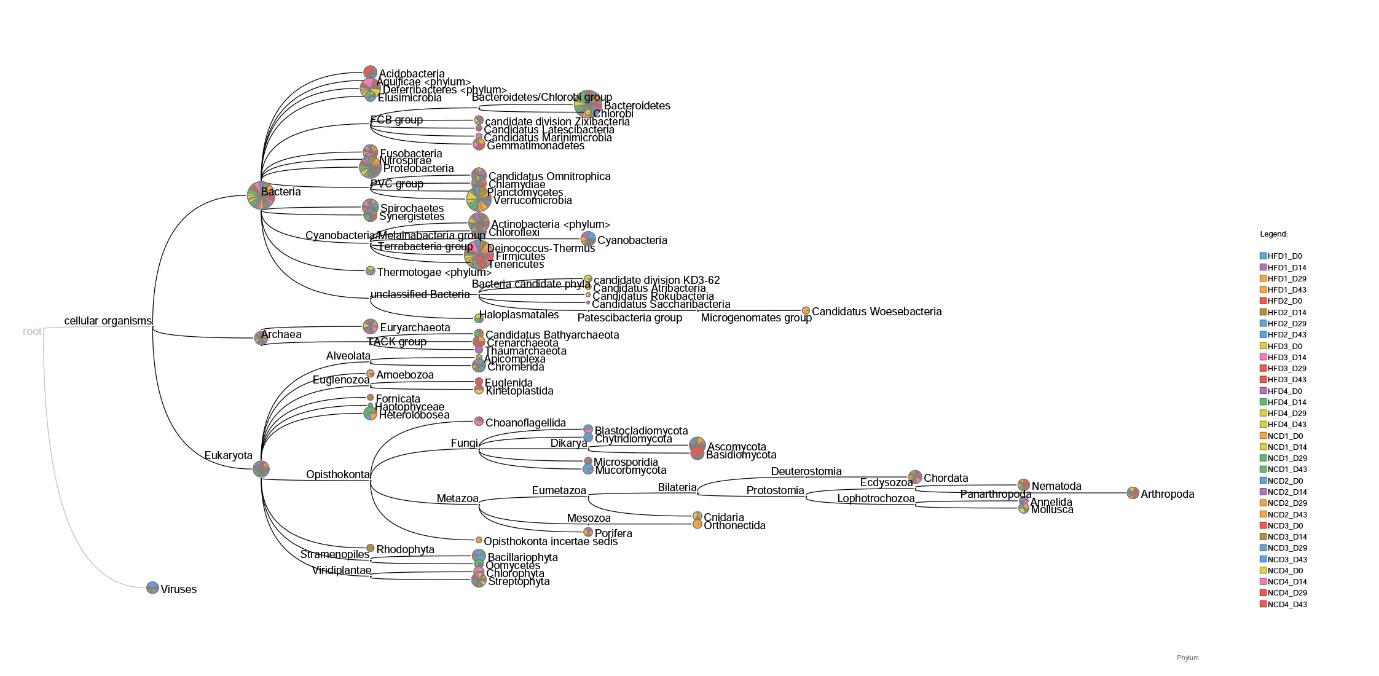
 Figure S3. The quantitative profile of the phylogenetic tree dataset. This figure was generated by MEGAN Community Edition (version 6.10.0) through importing the .biom format result outputted by MetaLab.


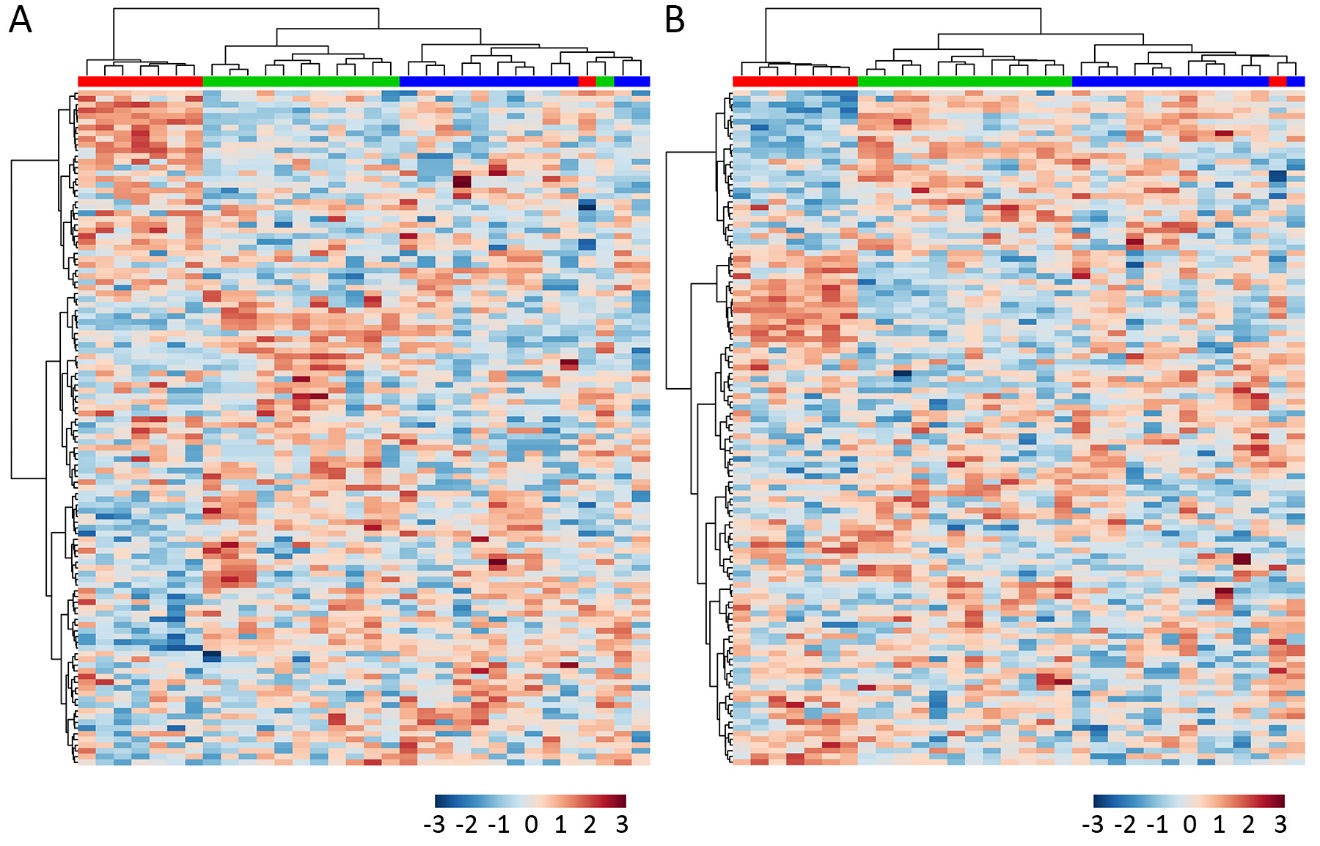


Figure S4. Heat map of (A) peptide counts; (B) spectral counts of bacterial species in 32 samples. Hierarchical row clustering was performed by Log_10_ (peptide count) or Log_10_ (spectral count), respectively. Red: initial condition; blue: LFD feeding; green: HFD feeding.


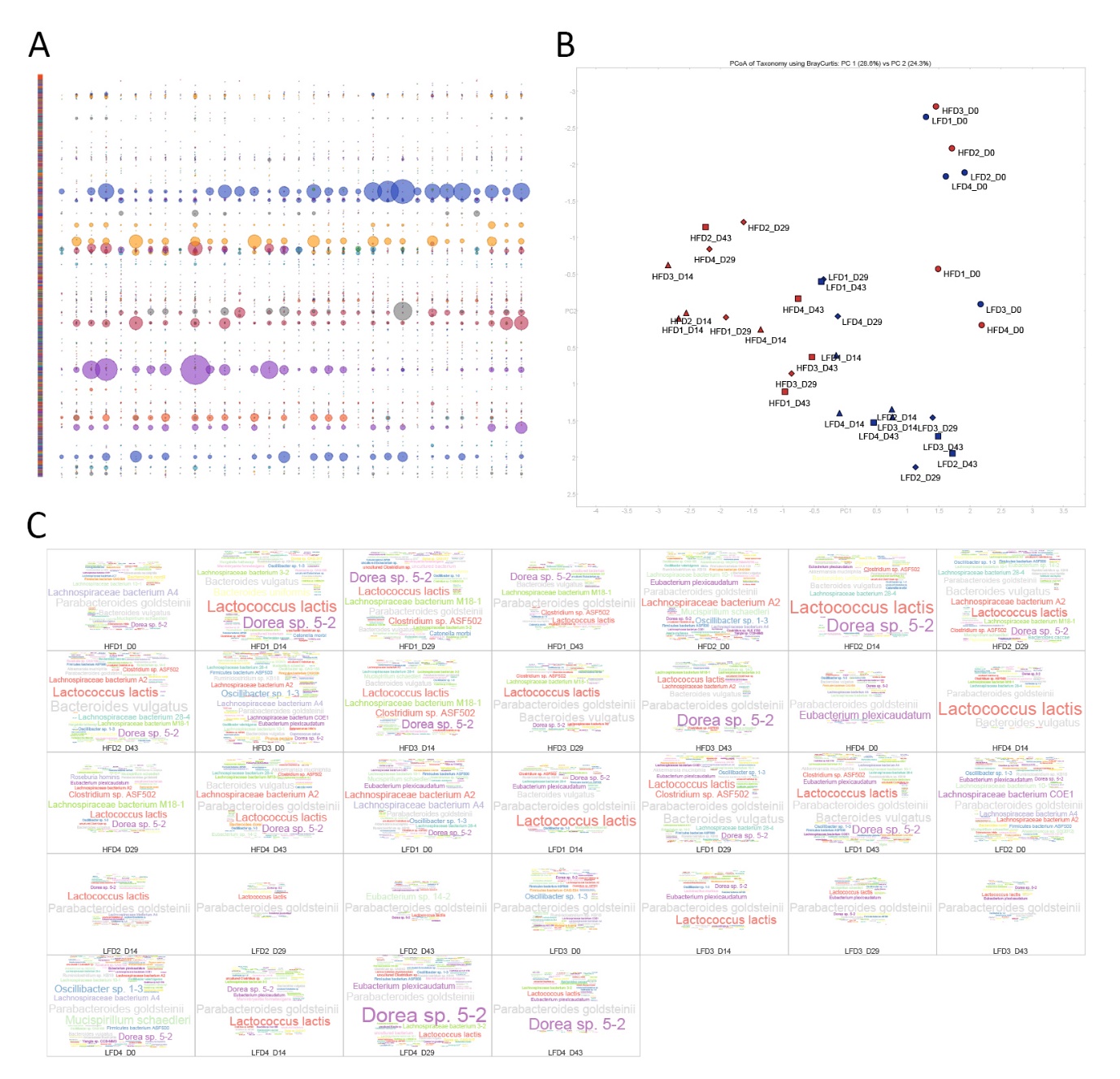


Figure S5. Charts illustrate the taxonomy profiles between different samples. Through importing the .biom file generated by MetaLab to MEGAN, various charts are visualized: (A) bubble chart, the columns represent different samples and each row represent one species, the abundance of species is reflected by the size of bubbles; (B) PCoA plot, the name of each spot is consisted with diet type, sample ID and days of feed, e.g., HFD2_29 means mouse ID is 2, feed with high-fat diet for 29 days; (C) word cloud chart, big words represent dominant species in this sample.


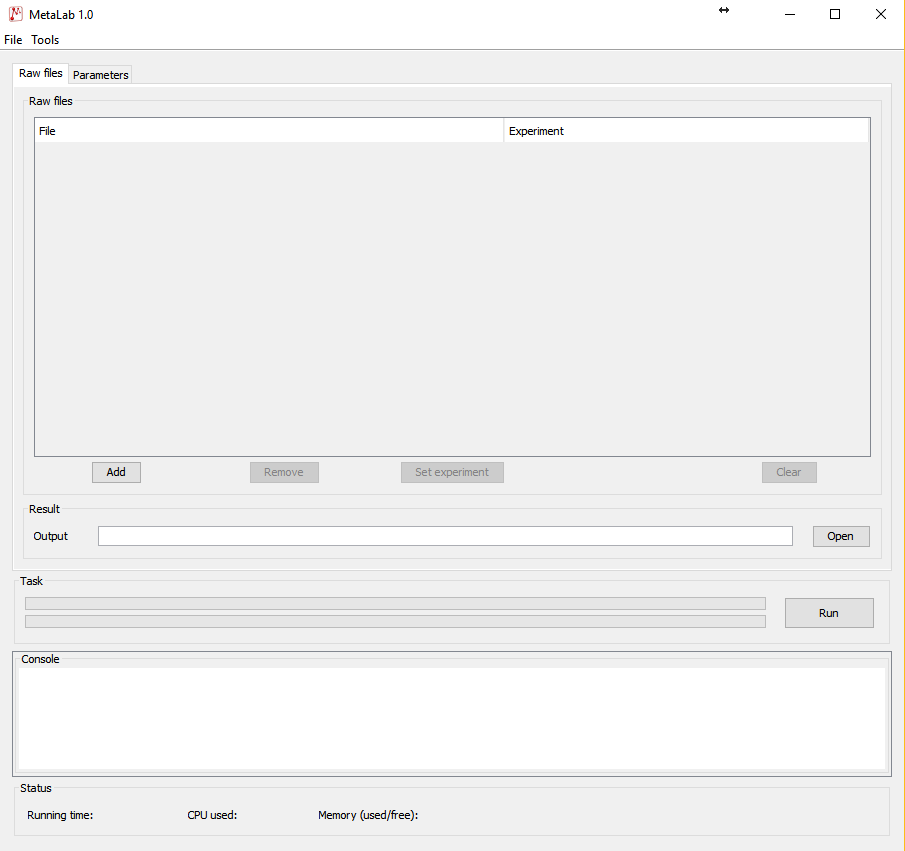


Figure S6. The main panel of MetaLab.


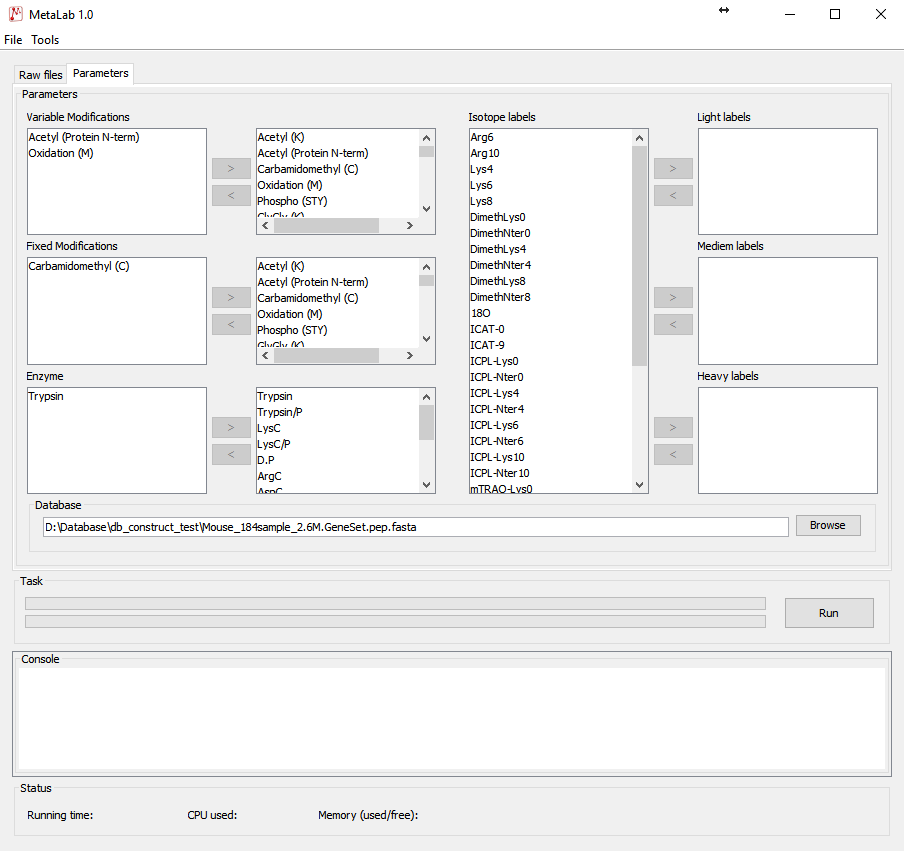


Figure S7. The parameter panel of MetaLab.


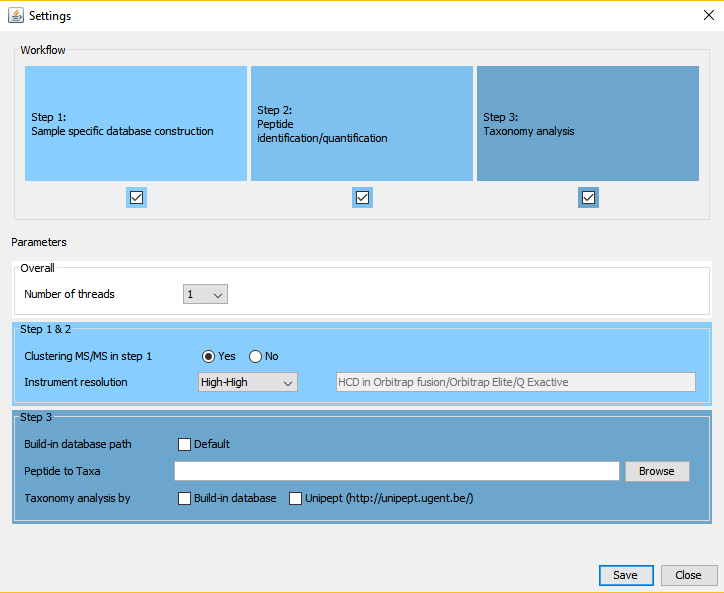


Figure S8. The workflow customization panel of MetaLab.
